# Application of Video Head Impulse Test in the Diagnosis and Follow-Up of Vestibular Schwannoma: Case Series, Narrative Literature Review and Clinical Practice Implications

**DOI:** 10.3390/jcm14207222

**Published:** 2025-10-13

**Authors:** Agnieszka Jasińska-Nowacka, Patrycja Torchalla, Tomasz Wojciechowski, Kazimierz Niemczyk

**Affiliations:** 1 Department of Otorhinolaryngology, Head and Neck Surgery, Medical University of Warsaw, 02-097 Warsaw, Poland; patrycja.torchalla@wum.edu.pl (P.T.); tomasz.wojciechowski@wum.edu.pl (T.W.); kniemczyk@wum.edu.pl (K.N.); 2 Department of Descriptive and Clinical Anatomy, Medical University of Warsaw, 02-097 Warsaw, Poland

**Keywords:** vestibular schwannoma, vertigo, video Head Impulse Test

## Abstract

**Background/Objectives:** Vestibular schwannoma (VS) is a benign cerebellopontine angle tumor causing audiological and vestibular symptoms. This pilot study aimed to describe the application of video Head Impulse Test (vHIT) in the diagnosis and follow-up of patients with unilateral VS treated surgically. The objective was to describe a detailed interpretation of vHIT—not only numerical parameters such as gain and corrective saccades, but also a visual analysis of vHIT curves. **Methods:** The results were presented in four cases for better understanding and more straightforward explanation. The patients underwent surgery through the middle cranial fossa and translabyrinthine approach. In each patient, vHIT examinations were performed preoperatively and at one month, three months, and one year after the surgery. **Results:** Before treatment, vestibular loss features varied within the presented cases. Findings of vestibulo-ocular reflex deficiency were most pronounced in the lateral semicircular canals. After the surgery, severe signs of acute labyrinth denervation were found during the first follow-up visit. Over time, features indicating central compensation became more pronounced, despite a decrease in gain in subsequent vHIT examinations. **Conclusions:** Detailed analysis of vHIT curves is crucial to analyze vestibulo-ocular reflex in patients with VS. Our preliminary data confirms that vHIT examination can be helpful in the postoperative follow-up assessment and compensation evaluation.

## 1. Introduction

The vast majority of cerebellopontine angle tumors are benign, with 80–90% being vestibular schwannomas (VSs), which originate from Schwann cells surrounding the eighth cranial nerve [1,2,3,4,5]. Despite their benign histological character, VSs can manifest as significant and disabling audio-vestibular symptoms, growing within the internal auditory canal and then into the cerebellopontine angle [1,6].

The most common clinical presentation of VS is unilateral progressive hearing loss (94% of patients) and subjective aural symptoms like tinnitus (83% of patients) [1,2,3]. Up to 75% of patients with VS may present balance disorders, including acute vertigo, chronic dizziness, and postural instability [6,7]. On the other hand, VS rarely presents as an acute vertigo episode, as its slow growth enables the gradual engagement of central adaptive processes known as vestibular compensation. Thus, patients more often report subtle, mild to moderate chronic balance problems. Consequently, vestibular symptoms may go unrecognized during the medical history-taking process. Still, patients may deny any subjective symptoms related to the balance system altogether, and comprehensive diagnostic tests are required to diagnose vestibular system impairment [8,9].

To objectively and quantitatively evaluate the vestibulo-ocular reflex, a video system measuring eye velocity during head rotation, known as the video head impulse test (vHIT), can be utilized [10,11,12]. The principal advantage of this method is its ability to assess all six semicircular canals—the anterior (ASC) and lateral canals (LCS), innervated by the superior vestibular nerve, and the posterior canal (PSC), innervated by the inferior branch. This is particularly relevant for lesions typically arising from the inferior vestibular nerve, such as VS.

This pilot study aimed to describe the application of vHIT in the diagnosis and postoperative follow-up of VS. The objective was to present a detailed interpretation of vHIT results in patients treated for unilateral VS, before and after the surgery. For better understanding and a more straightforward explanation, the results were presented in four example cases, focusing on the precise interpretation of the graphical representation.

## 2. Materials and Methods

### 2.1. Ethical Consideration

The local Ethics Committee approved the study protocol at the institution where the study was conducted (AKBE/203/2022). As this study includes a retrospective presentation of patients’ test results, no informed consent was required.

### 2.2. Patient Clinical Characteristics and Study Protocol

This study presents four patients to illustrate the vHIT protocol and provides a detailed analysis and interpretation of pre- and postoperative results. To illustrate the variability of vHIT results before and after surgery, four patients with different sizes of tumors were chosen for this case series. Moreover, uniform postoperative follow-up intervals were the criterion as well. Patients with prior medical history of vs. treatment were not included in this study. All patients were diagnosed with unilateral vs. visualized with the gadolinium-enhanced magnetic resonance imaging (MRI) of the posterior cranial fossa. Tumors were assessed according to the Koos grading scale [13]. Before the surgery, patients underwent ENT-otoneurological examination. A set of audiological and vestibular diagnostic tests was performed as described in a previous study [8]. The vHIT examinations were performed before surgery, one month after surgery, three months after surgery, and one year after surgery.

### 2.3. Surgical Treatment

The patients underwent tumor resection performed by the same experienced otosurgeon (one of the authors of the manuscript, K.N.).

In patients #1 and #3, the middle cranial fossa approach was performed, while the translabyrinthine approach was chosen in cases #2 and #4. The histopathological examination confirmed the presence of VS.

### 2.4. Video Head Impulse Test Protocol

The Video Head Impulse Test (vHIT) examination followed a standard protocol [8,9,10]. The examination was performed using the EyeSeeCam device (Interacoustics). During the test, patients were seated in front of a wall at a distance of approximately 1.2 m and were asked to keep their eyes focused straight ahead on a single point (a blue dot). The examiner performed fast movements of the patient’s head. The vHIT examination included a protocol that evaluated all six semicircular canals in three planes: the horizontal plane for the lateral canals, a plane oriented along the right-anterior-left-posterior (RALP) canals, and a plane oriented along the left-anterior-right-posterior (LARP) canals. Additionally, a suppression head impulse paradigm (SHIMP) was used to evaluate inhibition of the vestibulo-ocular reflex in the horizontal plane. In each semicircular canal plane, 15 technically correct trials were performed for each side.

The vestibulo-ocular reflex was assessed in the horizontal plane using the ratio of eye velocity to head velocity (gain), with results ranging from 0.8 to 1.2 considered normal. The symmetry was also analyzed, with interaural asymmetry rates of up to 25% accepted. Then, the presence and character of corrective catch-up saccades were evaluated. Saccades were categorized as overt, if present after the head impulse, and covert, if occurring during the head movement. Moreover, in results with both overt and covert saccades, the visual organization of saccades was analyzed, distinguishing between scattered and gathered patterns [11,12].

## 3. Results

### 3.1. Patients’ Clinical Data and Video Head Impulse Test Results

**Figure 1:**
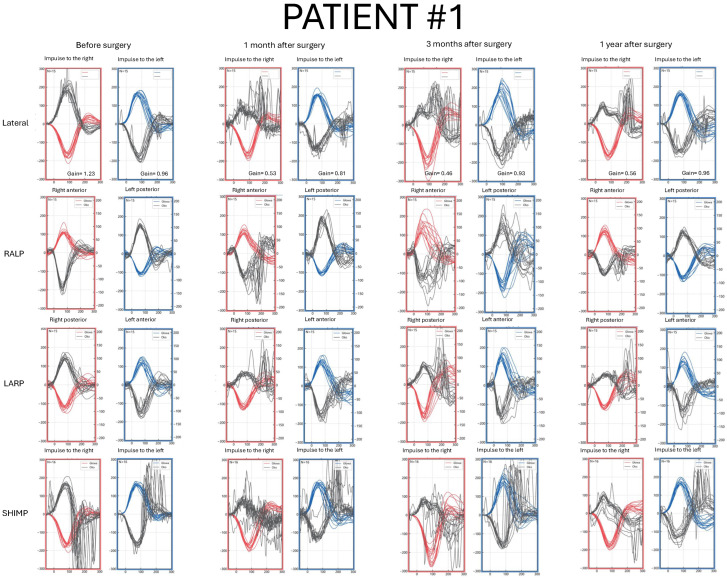
Video Head Impulse Test (vHIT) results in Patient #1 with a right-sided vestibular schwannoma, recorded before surgery and at 1, 3, and 12 months postoperatively. Red curves represent head movements to the right, blue curves represent head movements to the left, and black curves represent eye movements. LARP—left anterior, right posterior canal; RALP—right anterior, left posterior canal; SHIMP—suppression head impulse test.

**Figure 2:**
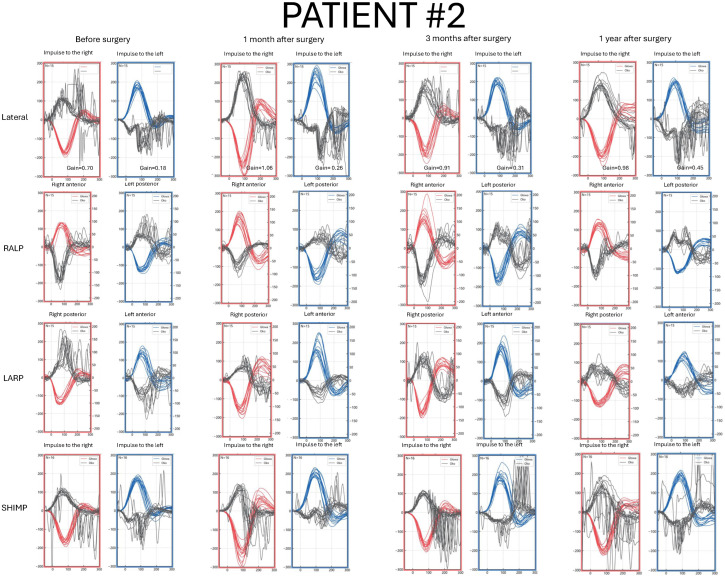
Video Head Impulse Test (vHIT) results in Patient #2 with a left-sided vestibular schwannoma, recorded before surgery and at 1, 3, and 12 months postoperatively. Red curves represent head movements to the right, blue curves represent head movements to the left, and black curves represent eye movements. LARP—left anterior, right posterior canal; RALP—right anterior, left posterior canal; SHIMP—suppression head impulse test.

**Figure 3:**
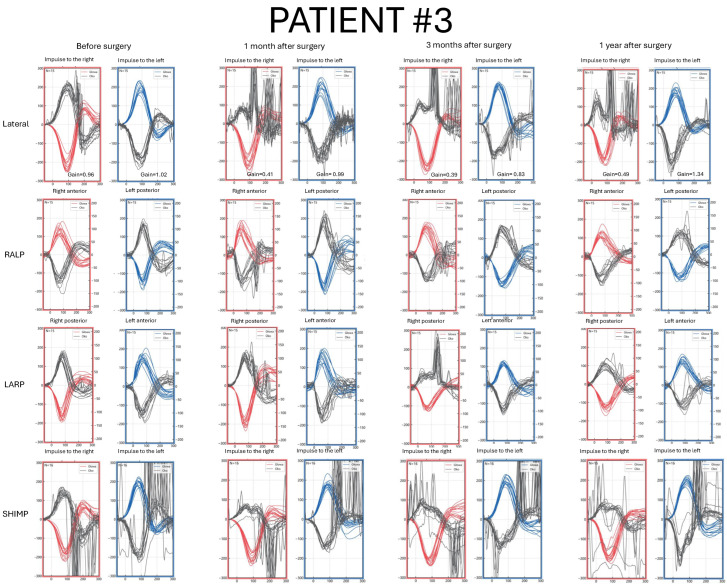
Video Head Impulse Test (vHIT) results in Patient #3 with a right-sided vestibular schwannoma, recorded before surgery and at 1, 3, and 12 months postoperatively. Red curves represent head movements to the right, blue curves represent head movements to the left, and black curves represent eye movements. LARP—left anterior, right posterior canal; RALP—right anterior, left posterior canal; SHIMP—suppression head impulse test.

**Figure 4:**
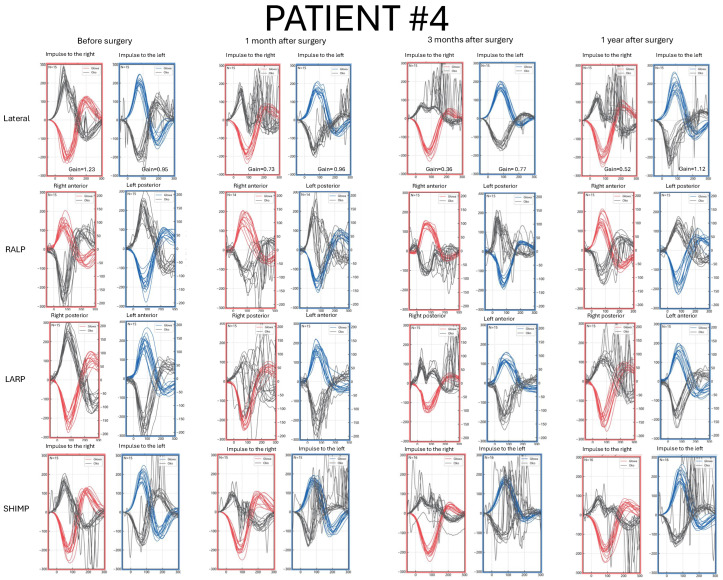
Video Head Impulse Test (vHIT) results in Patient #4 with a right-sided vestibular schwannoma, recorded before surgery and at 1, 3, and 12 months postoperatively. Red curves represent head movements to the right, blue curves represent head movements to the left, and black curves represent eye movements. LARP—left anterior, right posterior canal; RALP—right anterior, left posterior canal; SHIMP—suppression head impulse test.

Detailed pre- and postoperative vHIT results parameters are presented in Figure 1, Figure 2, Figure 3 and Figure 4 and Table S1.

### 3.2. Patient #1—Clinical Characteristics and vHIT Results

**Figure 5:**
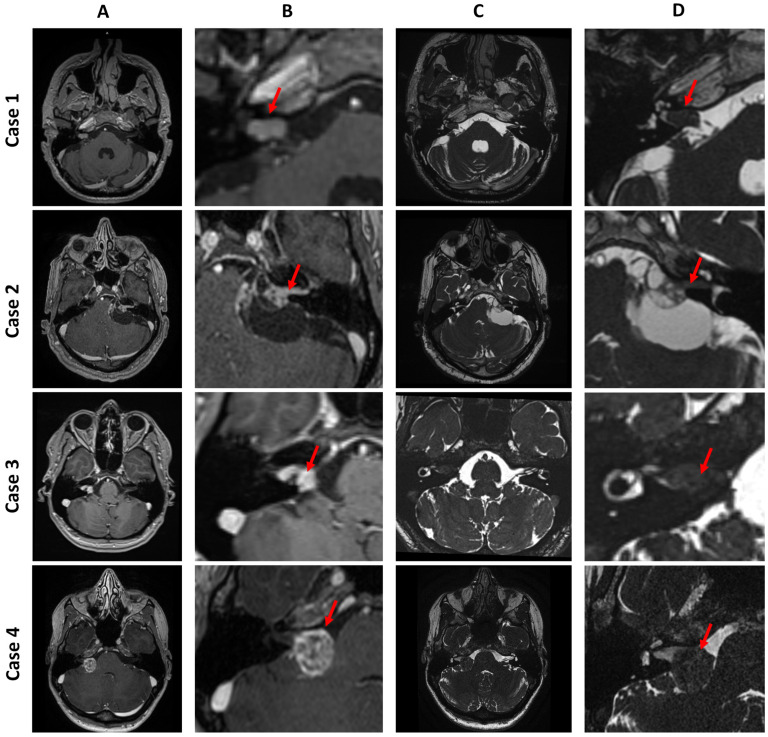
Four cases of vestibular schwannoma presented in four consecutive rows. Column (**A**) and column (**B**) show a general and detailed view of T1-weighted images with contrast enhancement. Column (**C**) and column (**D**) show a general and detailed view of T2-weighted images. The red arrow points to the tumor.

A 52-year-old male with a VS size of 12.5 mm × 6.5 mm × 5.5 mm on the right side (Figure 5). First symptoms were unilateral hearing deterioration on the right side, with deterioration in speech recognition. No additional symptoms were reported (Table S1).

Before surgery, LSC gain was normal, and no corrective saccades were found on the affected side. Several overt saccades with small amplitude were present on the contralateral side. Results in the RALP/LARP planes were normal. Physiological saccades were present in the SHIMP protocol (Figure 1).

One month after surgery, a gain reduction and overt and covert saccades in a scattered pattern in the LSC on the tumor’s side were found. In ACS and PSC, covert and overt saccades were present, respectively. Three months after the surgery, saccade latency was shorter, with a greater number of covert peaks. vHIT performed one year after the surgery showed a narrow range of saccade latencies, with the majority occurring within the time of the head movement. LSC gain remained reduced on the affected side. No consistent, clinically relevant saccades were detected in the SHIMP after surgery (Figure 1).

### 3.3. Patient #2—Clinical Characteristics and vHIT Results

A 68-year-old male with a VS size of 40 mm × 30 mm × 24 mm on the left side (Figure 5). The first symptom was unilateral tinnitus. After diagnosis, hearing loss on the left side, dizziness, and headache in the occipital region appeared before tumor removal (Table S1).

In the preoperative vHIT examination, on the tumor’s side, numerous covert and overt saccades in each of the semicircular canals and LSC gain reduction were observed. On the contralateral side, saccades were also visualized, and LSC gain was below the normal value but higher compared with the affected side. The SHIMP protocol result was abnormal while turning the head to the affected side (Figure 2).

After the surgery, LSC gain on the affected side remained reduced, but showed a gradual increase over time. The LSC gain value on the healthy side was within normal range. With subsequent follow-up examinations, covert saccades became more frequent (Figure 2). 

### 3.4. Patient #3—Clinical Characteristics and vHIT Results

A 39-year-old female with a VS size of 14 mm × 8.7 mm × 7 mm on the right side (Figure 5). The first symptom was dizziness. Hearing deterioration in the right ear and episodes of spinning vertigo were reported by the patient prior to the surgery (Table S1).

Before the surgery, LSC gain was normal and symmetrical; still, numerous covert catch-up saccades were visible on the tumor’s side. Results in RALP/LARP planes and SHIMP protocol were normal (Figure 3).

After the surgery, LCS decreased gain, and covert and overt saccades in a gathered pattern were observed in each follow-up vHIT. Analyzing anterior and posterior semicircular canals, saccade latency and amplitude became progressively smaller over time. In the SHIMP protocol, physiological saccades were still visible on the early follow-up visit, and by the one-year follow-up, no clinically significant peaks were observed (Figure 3).

### 3.5. Patient #4—Clinical Characteristics and vHIT Results

A 38-year-old female with a VS size of 19 mm × 16 mm × 13 mm on the right side (Figure 5). The first symptom was sudden hearing loss on the right side. Tinnitus, vertigo, dizziness, and headache on the right side were reported prior to surgical treatment (Table S1).

In the preoperative vHIT result, covert saccades superimposed on the plotted curves were observed symmetrically in the lateral plane examination. LSC gain value was within the normal range. Subtle, covert corrective saccades were also visible in the ASC and PSC results (Figure 4).

Postoperatively, catch-up saccades were visible in all canals on the affected side in each follow-up vHIT, with an increasing number of covert saccades over time. LSC gain in basic and SHIMP protocols remained reduced after surgery; however, some saccades were visible in the SHIMP while turning the head to the right in a one-year follow-up (Figure 4).

Case 1 is a Koos I intracanalicular vestibular schwannoma in the right internal acoustic meatus with CSF cap separating the tumor from the cochlea.
Case 2 is a Koos IV vestibular schwannoma with a cystic component in the left cerebellopontine angle, causing brainstem displacement.
Case 3 is a Koos II vestibular schwannoma occupying the fundus of the right internal acoustic meatus and protruding into the cerebellopontine angle.
Case 4 is a Koos III vestibular schwannoma present in the right cerebellopontine angle cistern, occupying less than half of the internal acoustic meatus.

## 4. Discussion

In patients with VS, vestibular complaints are less frequently reported than subjective hearing loss and tinnitus [1,2,6,9]. Patients do not present with a typical, uniform neurotological symptom pattern. The progressive, gradual vestibular loss is rarely the most prominent symptom, which makes it easily overlooked during history taking. In consequence, they are presumed to be underestimated. Then, as the tumor grows, balance disorders become disabling in some patients and significantly affect the quality of life in patients with unilateral vestibular schwannoma [14].

Although MRI remains the gold standard in diagnosing cerebellopontine angle tumors, otoneurological diagnostic tests are necessary for detailed evaluation of the vestibular system in patients with VS [4,9]. Despite the evolution and improvement in vestibular diagnostic methods over the past two decades, videonystagmography (VNG) remains the most popular method for evaluating the vestibulo-ocular reflex. Still, it must be noticed that caloric tests evaluate the lateral semicircular canal innervated by the superior vestibular branch. In evaluating the inferior vestibular nerve, cervical vestibular evoked myogenic potentials (cVEMPs) and vHIT are presumed to be more specific [15,16,17,18,19,20,21,22,23,24]. However, inconsistent conclusions are reported in the literature, mainly due to variations in the vHIT evaluation protocol [16,17]. As described by West et al. [17], the vHIT sensitivity for detecting VS was 80%, and there was a significant correlation between vHIT gain and saccades and caloric responses. Moreover, a relationship between hearing loss and vestibulo-ocular reflex gain has been described [18].

While hearing loss occurs in VS regardless of tumor size, studies have reported a correlation between tumor grade and vestibular dysfunction [19]. In particular, the relationship with vHIT results is important in the context of the present study. Interestingly, in the study by West et al. [17], medium-sized VS were characterized by the highest gain asymmetry, while larger tumors were associated with numerous catch-up saccades. In the present case series, Patient #1, with a small intrameatal tumor, exhibited normal gain and no corrective saccades before surgery. At the same time, vestibulo-ocular reflex abnormalities were found in patients with more advanced lesions. In cases #3 and #4, preoperative vHIT demonstrated covert saccades and gain within the normal range.

In contrast, numerous overt catch-up saccades and decreased gain were observed before surgery in Patient #2, which corresponded with the tumor size. Consistent with our preliminary reports, corrective saccades with normal gain value were observed in the VS cohort analyzed by Picciotti et al. [20]. The results are relevant in daily clinical practice, as they emphasize the morphological analysis of vHIT curves with detailed covert saccade detection. Evaluating vHIT with a gain parameter only results in significantly lower sensitivity in vs. diagnosis [16,21]. Recently, Jencen et al. [22] introduced a new vHIT parameter, evaluating physiological saccades in the SHIMP—the anticompensatory saccade amplitude ratio. Authors reported that analyzing vestibulo-ocular reflex in both standard and suppression protocols improves the sensitivity of vHIT in VS diagnosis.

Taking into consideration the tumor’s origin in the inferior vestibular branch, we presumed that RALP and LARP planes should be most specific and sensitive in early VS diagnosis. Surprisingly, in Patients #3 and #4, covert saccades were present in the LSC only, while the curves in the RALP and LARP planes were standard. That observation is consistent with our previous study [9]. We hypothesized that the slow growth of VS arising from the inferior vestibular branch may preserve its function; however, compression of the superior nerve can lead to injury of the peripheral fibers. However, a recent study analyzing 19 patients with vs. concluded that in roughly half the cases (52.6%), both superior and inferior vestibular branch impairment were found in preoperative vHIT [23]. Similarly, Taylor et al. [24] reported that dysfunction of the inferior and superior vestibular nerves develops in parallel, while isolated impairment of the inferior branch was found in 10.4% of patients. On the other hand, Fujiwara et al. [25] analyzed 15 patients with vs. and noticed that ASC deficit occurs significantly less frequently than LSC and PSC dysfunction.

Depending on the tumor size, growth rate, and other clinical factors, watchful observation, radiotherapy, or surgical treatment may be recommended. In patients qualified for surgical treatment, the middle fossa, translabyrinthine, or retrosigmoidal approach can be chosen. Surgical tumor removal results in vertigo due to the complete vestibular denervation, which manifests as an acute vestibular episode caused within the first postoperative days. A detailed analysis of postoperative vHIT results in patients after vs. surgery was described by Pogson et al. [26]. In that study, first and second catch-up saccades in each trial were analyzed. The authors suggested that the first signs of compensation can be found in the vHIT result one week after vestibule deafferentation. Moreover, they observed that the frequency and amplitude of the second saccade decreased over time, while characteristics of the first saccade remained unchanged in a one-year follow-up. A difference in postoperative saccade characteristics was described between the semicircular canals [23]. Regarding these observations, normalization of the ASC result was observed in Patient #3 one year after surgery in our study.

Slow growth of the tumor permits gradual compensation for progressive nerve fiber compression and damage. Nevertheless, in patients with partially preserved vestibular function, surgical treatment may sometimes disturb this balance. In consequence, acute spinning vertigo immediately after vs. surgery is a common complaint, and its severity is related to the preoperative responsiveness of the labyrinth, the clinical course of the disease, and the tumor growth rate and dynamics. After iatrogenic vestibular denervation, the implementation of central adaptive mechanisms leads to vestibular and balance compensation. Spontaneous functional improvement is expected to occur within the first three months after vs. surgery [9]. Nevertheless, prolonged postural imbalance is reported by 10% to 78% patients [27,28]. Despite numerous studies, clinical predictors of persistent postoperative disequilibrium remain poorly understood. Thus, a detailed analysis of innovative vestibular test results before and after the surgery would be crucial in better understanding vestibular compensation mechanisms. Another parameter of corrective saccades that may be valuable in postoperative compensation assessment is their organization [11,12]. Interestingly, a random pattern is correlated with incomplete subjective compensation, as measured by the DHI score, one year after vs. surgery [29]. In the present case series, saccades occurred randomly in one-year follow-up in patients with more advanced tumors after the TL approach (cases #2 and #4). Further studies on a larger group of patients are necessary to verify if the saccade organization pattern is related to the tumor size. Taking into consideration the availability of vHIT test and good patient tolerance, we believe this method can serve as a useful tool in monitoring vestibular function and evaluating the compensation process in patients with VS. It could also be helpful in identifying patients requiring rehabilitation and personalizing postoperative care.

### Study Limitations

The primary study limitation is a small patient group. However, we aimed to focus on detailed vHIT interpretation based on the visual assessment of the curves, in contrast to studies analyzing gain value and quantitative numerical saccade parameters. Thus, we decided to present an analysis illustrated with case examples. Further studies on a larger group of patients are necessary to obtain reliable data on vestibular compensation depending on the clinical course and treatment method.

## 5. Conclusions

Modern vestibular tests are helpful in diagnosing cerebellopontine angle tumors. They can determine VS laterality and the precise receptors affected by the tumor. In the present pilot study, example cases demonstrated that vHIT examination enables a detailed analysis of the function of all six semicircular canals; thus, the method can reveal the involvement of the vestibular receptors innervated by the superior and inferior vestibular branches. The primary conclusion of the present study is that, despite originating from the inferior vestibular nerve, cerebellopontine angle tumors can affect the function of the anterior and lateral semicircular canals. Both gain reduction and corrective saccades can be found in patients with VS, as visualized in our findings. A detailed analysis of vHIT curves is crucial for evaluating the vestibulo-ocular reflex in patients with VS. Furthermore, our preliminary data suggest that vHIT examination can be helpful in postoperative follow-up assessments and compensation evaluations. We believe it can lead to personalized postoperative vestibular rehabilitation in patients with VS. A set of vestibular tests planned pre- and postoperatively would help in better understanding the vs. pathophysiology. Nowadays, with several commercial vHIT devices available and costs relatively low compared to other vestibular tests, the method is becoming increasingly accessible.

## Data Availability

The Dataset is available on request from the authors.

## Supplementary Materials

The following supporting information can be downloaded at: https://www.mdpi.com/article/10.3390/jcm14207222/s1, Table S1: Video Head Impulse Test results obtained before the surgical removal of the vestibular schwannoma, one month, three months, and one year after the surgery in Patients 1–4, respectively.
