# Supplementary material for: Application of Video Head Impulse Test in the Diagnosis and Follow-Up of Vestibular Schwannoma: Case Series, Narrative Literature Review and Clinical Practice Implications
Source: J Clin Med. 2025 Oct 13;14(20):7222. doi: 10.3390/jcm14207222 (PMC12565445; doi:10.3390/jcm14207222)
Supplement: Supplementary file 1 [file jcm-14-07222-s001.zip › jcm-3871455-supplementary.pdf]

Table S1. Video Head Impulse Test results obtained before the surgical removal of the vestibular schwannoma, one month, three months and one year after the surgery in Patients 1-4, respectively.

**PATIENT #1**				
AGE<br>52	FIRST SYMPTOM<br>hearing deterioration	TUMOR SIDE<br>right	TUMOR SIZE IN MRI<br>12,5x 6,5x 5,5 mm	SURGICAL APPROACH<br>MFA
SCC/ TIME	BEFORE SURGERY	1 MONTH<br>AFTER SURGERY	3 MONTHS<br>AFTER SURGERY	1 YEAR<br>AFTER SURGERY
TUMOR SIDE				
LATERAL	no corrective saccades<br>gain= 1,23	covert and overt saccades<br>(scattered pattern)<br>gain= 0,53	overt and covert saccades<br>(scattered pattern)<br>gain= 0,46	overt saccades<br>gain= 0,56
ANTERIOR	no corrective saccades	covert saccades	no correctives saccades; artifacts	covert and overt saccades<br>(scattered pattern)
POSTERIOR	no corrective saccades	overt saccades	overt saccades	overt saccades
SHIMP	corrective saccades<br>gain= 1,08	no corrective saccades<br>gain= 0,35	single corrective saccades<br>gain= 0,39	single corrective saccades<br>gain= 0,54
HEALTHY SIDE				
LATERAL	small amplitude overt saccades<br>gain= 0,96	overt saccades<br>gain= 0,81	no correctives saccades<br>gain= 0,93	overt saccades<br>gain= 0,96
ANTERIOR	no corrective saccades	no corrective saccades	no correctives saccades; artifacts	no correctives saccades
POSTERIOR	no correctives saccades	no corrective saccades	no correctives saccades; artefacts	no correctives saccades
SHIMP	corrective saccades<br>gain= 0,96	corrective saccades<br>gain= 0,76	corrective saccades<br>gain= 0,90	corrective saccades<br>gain= 0,80
PATIENT #2				
AGE<br>68	FIRST SYMPTOM<br>tinnitus	TUMOR SIDE<br>left	TUMOR SIZE IN MRI<br>40x 30x 24 mm	SURGICAL APPROACH<br>TL
SCC/ TIME	BEFORE SURGERY	1 MONTH<br>AFTER SURGERY	3 MONTHS<br>AFTER SURGERY	1 YEAR<br>AFTER SURGERY
TUMOR SIDE				
LATERAL	covert and overt saccades<br>(scattered pattern)<br>gain= 0,18	covert and overt saccades<br>(gathered pattern)<br>gain= 0,26	covert saccades<br>gain= 0,31	overt and covert saccades<br>(scattered pattern)<br>gain= 0,45
ANTERIOR	covert saccades	no corrective saccades	no corrective saccades	no correctives saccades
POSTERIOR	covert saccades	no corrective saccades	no corrective saccades	no correctives saccades
SHIMP	no corrective saccades<br>gain= 0,24	single corrective saccades<br>gain= 0,19	corrective saccades<br>gain= 0,26	no correctives saccades<br>gain= 0,43
HEALTHY SIDE				
LATERAL	covert saccades	overt saccades	no correctives saccades	no correctives saccades

	antisaccades<br>gain= 0,70	gain= 1,06	gain= 0,91	gain= 0,98
ANTERIOR	no corrective saccades	no corrective saccades	no corrective saccades	no corrective saccades
POSTERIOR	no corrective saccades	no corrective saccades	no corrective saccades	no corrective saccades
SHIMP	corrective saccades<br>gain= 0,68	single corrective saccades<br>gain= 0,61	corrective saccades<br>gain= 0,61	corrective saccades gain= 0,58
PATIENT #3				
AGE<br>39	FIRST SYMPTOM<br>dizziness	TUMOR SIDE<br>right	TUMOR SIZE IN MRI<br>14x 8,7x 7 mm	SURGICAL APPROACH<br>MFA
SCC/ TIME	BEFORE SURGERY	1 MONTH<br>AFTER SURGERY	3 MONTHS<br>AFTER SURGERY	1 YEAR<br>AFTER SURGERY
TUMOR SIDE				
LATERAL	covert saccades<br>gain= 0,96	covert and overt saccades<br>(gathered pattern)<br>gain= 0,41	covert and overt saccades<br>(gathered pattern)<br>gain= 0,39	covert and overt saccades<br>(gathered pattern)<br>gain= 0,49
ANTERIOR	no corrective saccades	covert saccades	overt saccades	no corrective saccades
POSTERIOR	no corrective saccades	overt saccades	covert saccades	no corrective saccades
SHIMP	corrective saccades<br>gain= 0,84	corrective saccades<br>gain= 0,44	corrective saccades<br>gain= 0,34	no corrective saccades<br>gain= 0,26
HEALTHY SIDE				
LATERAL	no corrective saccades<br>gain= 1,02	no corrective saccades<br>gain= 0,99	covert saccades<br>gain= 0,83	no corrective saccades<br>gain= 1,34
ANTERIOR	no corrective saccades	single overt saccades	no corrective saccades	no corrective saccades
POSTERIOR	no corrective saccades	no corrective saccades	no corrective saccades	no corrective saccades
SHIMP	corrective saccades<br>gain= 0,90	corrective saccades<br>gain= 0,83	corrective saccades<br>gain= 0,75	corrective saccades gain= 0,83
PATIENT #4				
AGE<br>38	FIRST SYMPTOM<br>Hearing deterioration	TUMOR SIDE<br>right	TUMOR SIZE IN MRI<br>19x 16x 13 mm	SURGICAL APPROACH<br>TL
SCC/ TIME	BEFORE SURGERY	1 MONTH<br>AFTER SURGERY	3 MONTHS<br>AFTER SURGERY	1 YEAR<br>AFTER SURGERY
TUMOR SIDE				
LATERAL	covert saccades<br>gain= 1,23	covert and overt saccades<br>(scattered pattern)<br>gain= 0,73	covert and overt saccades<br>(scattered pattern)<br>gain= 0,36	covert saccades<br>gain= 0,54
ANTERIOR	covert saccades	covert and overt saccades<br>(scattered pattern)	overt saccades	overt and covert saccades<br>(scattered pattern)<br>artifacts
POSTERIOR	covert saccades	covert and overt saccades<br>(scattered pattern)	overt saccades	overt and covert saccades<br>(scattered pattern)
SHIMP	corrective saccades<br>gain= 0,77	no corrective saccades<br>gain= 0,40	no corrective saccades<br>gain= 0,24	single corrective saccades<br>gain= 0,41
HEALTHY SIDE				

LATERAL	covert saccades, antisac-cades, artifacts<br>gain= 0,95	no corrective saccades<br>antisaccades<br>gain= 0,96	no corrective saccades<br>gain= 0,77	no correctives saccades<br>gain= 1,12
ANTERIOR	no corrective saccades	no corrective saccades<br>artifacts	no corrective saccades	no corrective saccades
POSTERIOR	no corrective saccades	no corrective saccades<br>artifacts	no corrective saccades	no corrective saccades<br>artifacts
SHIMP	corrective saccades<br>gain= 0,79	corrective saccades<br>gain= 0,82	corrective saccades<br>gain= 0,67	corrective saccades<br>gain= 0,70

SCC-semicircular canal.
